# The AlkB Family: Potential Prognostic Biomarkers and Therapeutic Targets in Glioblastoma

**DOI:** 10.3389/fonc.2022.847821

**Published:** 2022-03-10

**Authors:** Songshan Feng, Zhijie Xu, Jinwu Peng, Mingyu Zhang

**Affiliations:** ^1^Department of Neurosurgery, Xiangya Hospital, Central South University, Changsha, China; ^2^Department of Pathology, Xiangya Hospital, Central South University, Changsha, China; ^3^Department of Pathology, Xiangya Changde Hospital, Changde, China; ^4^National Clinical Research Center for Geriatric Disorders, Xiangya Hospital, Central South University, Changsha, China

**Keywords:** glioblastoma, AlkB family, patient prognosis, DNA damage repair, immune infiltration

## Abstract

The AlkB family of Fe (II) and α-ketoglutarate-dependent dioxygenases works by removing alkyl substituents from alkylation-damaged nucleic acid bases through oxidative dealkylation, subsequently affecting tumor progression and patient prognosis. However, the specific roles of the AlkB family in Glioblastoma remain to be elucidated. By taking advantage of the abundant bioinformatics databases, such as GEPIA2, cBioPortal and TIMER, we performed a comprehensive analysis of the AlkB family in GBM, and managed to identify the significant prognostic hallmarks and therapeutic targets within this family. We found that the expression levels of ALKBH2 and ALKBH8 were significantly up-regulated in GBM compared with normal tissues. Meanwhile, the patients with high levels of ALKBH2 and ALKBH8 possessed significant poor overall survival (OS). In addition, the results suggested that the biological function of the AlkB family was closely related to DNA damage repair, cell metabolism, cell proliferation and tumor immune infiltration in GBM. Furthermore, the high expression of ALKBH8 in GBM was verified by immunohistochemistry. Taken together, this study could provide meaningful information about the aberrant AlkB family associated with GBM initiation and progression, and help clinicians precisely predict patient survival and select alternative therapeutic drugs.

## Introduction

Glioblastoma (GBM) is the most common malignant primary tumor of the central nervous system, accounting for about 14.5% of all primary brain tumors and 48% of all malignant primary brain tumors (1). It has the characteristics of easy recurrence, high malignancy and poor prognosis (2, 3). In the United States, the average age-adjusted incidence of GBM is 3.21 per 100,000 population (1). GBM patients have a poor prognosis with a 5-year survival rate of 7.2% and a median survival of 8 months (1). The standard approach for GBM is maximal safe surgical resection followed by radiotherapy with concomitant temozolomide (TMZ, an alkylating agent) and a further 6 cycles of temozolomide (4). Even after the most effective treatment, the average survival time of GBM patients is only 14.5-16.6 months (5). The illustration of the molecular profile of GBM confers great significance to understand the occurrence and development of the disease. It was reported that patients with as isocitrate dehydrogenase 1 (IDH-1), IDH-2 mutation or MGMT methylation have relatively longer survival time (6, 7). However, we need to continue to depict the molecular features to combat this devastating disease.

The AlkB family of Fe (II) and α-ketoglutarate-dependent dioxygenases is a group of direct reversal DNA repair enzymes (8). The family of proteins works by removing alkyl substituents from alkylation-damaged nucleic acid bases through oxidative dealkylation (9, 10). So far, nine homologues (ALKBH1–8 and FTO) have been identified in human cells (11). The class of proteins possess a variety of biological functions, such as DNA repair, participating in the RNA metabolism, acting as demethylase or participating in fatty acid metabolism (12–14). And this family has been reported as prognostic biomarkers and therapeutic targets of different kinds of tumor, including head and neck cancer, ovarian serous carcinoma, lung adenocarcinoma, and colorectal cancer (15–18). In particular, ALKBH2 was discovered to play a significant role in TMZ and photodynamic therapy resistance in GBM (19, 20). However, the specific role of the AlkB family in GBM remains to be furtherly elucidated.

By taking advantage of the abundant bioinformatics databases (**Table 1**), we performed a comprehensive analysis of the AlkB family in GBM, and managed to identify the significant prognostic hallmarks and therapeutic targets within this family, which we believe could help clinicians precisely predict patient survival and select alternative therapeutic drugs.

**Table 1:** The online databases applied to evaluate the expression and biological functions of AlkB family in GBM.

Databases	Authors	Publication data	Samples	URL
GEPIA2	Tang et al. (21)	2019	Tissues	http://gepia2.cancer-pku.cn/#index
UALCAN	Chandrashekar et al. (22)	2017	Tissues	http://ualcan.path.uab.edu/index.html
PrognoScan	Mizuno et al. (23)	2009	–	http://dna00.bio.kyutech.ac.jp/PrognoScan/PrognoScan.html
cBioPortal	Cerami et al. (24)	2012	Tissues	http://cbioportal.org
STRING	Szklarczyk et al. (25)	2021	–	https://cn.string-db.org/
WebGestalt	Liao et al. (26)	2019	–	http://www.webgestalt.org/option.php
TIMER	Li et al. (27)	2017	Tissues	https://cistrome.shinyapps.io/timer/
LinkedOmics	Vasaikar et al. (28)	2018	Tissues	http://www.linkedomics.org/login.php

## Materials and Methods

### GEPIA2

GEPIA2 (Gene Expression Profiling Interactive Analysis, http://gepia2.cancer-pku.cn/#index) is a web resource based om tumor and normal samples from the TCGA and the GTEx databases, which provides differential gene expression profiling, correlation analysis, and patient survival analysis (21, 29). In this study, we utilized the “single gene analysis” module of GEPIA2 to compare mRNA expression differences between GBM and normal brain tissue. Student’s t test was used to generate p values for mRNA expression, and p < 0.05 was considered statistically significant.

### UALCAN

UALCAN (http://ualcan.path.uab.edu/index.html) is a convenient, interactive web-portal to perform analyses of TCGA gene expression data, which provides analyses of relative expression of genes across tumor and normal samples, estimation of the effect of gene expression level and clinicopathologic features on patient survival, and identification of the top up and down regulated genes in individual cancer types (22). In this research, the expression levels of the AlkB family were obtained through the “TCGA Gene analysis” module of the website. Differences in mRNA expression were calculated by Student’s t test, and p < 0.05 was considered statistically significant.

### The Human Protein Atlas

The Human Protein Atlas website (http://www.proteinatlas.org/) is an information database of protein expression patterns in normal human tissues and in cancer, which can identify clinically useful biomarkers by applying antibodies and protein expression data as tools (30). In this study, we obtained the protein expression profiles of the AlkB family members in normal brain and GBM tissues through this database.

### PrognoScan

PrognoScan (http://dna00.bio.kyutech.ac.jp/PrognoScan/PrognoScan.html) is a database offering a tool for assessing the relationship between gene expression and tumor patient prognosis, which employs the minimum P-value approach for grouping patients so as to find the optimal cut point in continuous gene expression measurement (23). In our study, we utilized this useful database to evaluate the relationship between the AlkB family gene expression and GBM patient overall survival.

### cBioPortal

The cBioPortal (cBio Cancer Genomics Portal, http://cbioportal.org) is a free resource for interactive exploration of multidimensional cancer genomics, through which we can get access to molecular profiles and clinical attributes from large-scale cancer genomics projects (24). In this study, we acquired the genetic alteration map of the AlkB family members in GBM tissues by searching this database.

### STRING

The STRING (https://cn.string-db.org/) is an open-access database providing integration of all known and predicted protein-protein interactions (PPIs), including physical interactions and functional associations, by automated text mining of the scientific literature, databases of interaction experiments, computational interaction predictions and systematic transfers of interaction evidence from one organism to another (25, 31). In our study, we utilized this database to analyze the interaction of altered proteins in tumor samples with mutation of at least one gene of AlkB family.

### Cytoscape

Cytoscape is an open software which can visually integrate biomolecular interaction networks with high-throughput expression data and other molecular states into a unified conceptual framework (32). In the study, we performed functional integration of 38 altered proteins in tumor samples with mutation of at least one gene of AlkB family.

### WebGestalt

WebGestalt (WEB-based Gene SeT AnaLysis Toolkit, http://www.webgestalt.org/option.php) is a functional enrichment analysis web tool, which supports three methods for enrichment analysis, including Over-Representation Analysis (ORA), Gene Set Enrichment Analysis (GSEA), and Network Topology-based Analysis (NTA) (26). In our study, we performed Gene ontology (GO) enrichment analysis by applying the WebGestalt website.

### TIMER

TIMER web server (https://cistrome.shinyapps.io/timer/) is a comprehensive resource for systematical analysis of immune infiltrates across diverse cancer types (27, 33). In this study, we utilized “gene module” to explore the correlation between AlkB family gene expression and abundance of immune infiltrates. And the “survival module” was applied to assess the association of clinical outcome with immune cell infiltration and the AlkB family gene expression. The multivariable cox proportional hazard model was used as the statistical method.

### LinkedOmics

LinkedOmics (http://www.linkedomics.org/login.php) is publicly available website which includes multi-omics data from all 32 TCGA Cancer types and 10 Clinical Proteomics Tumor Analysis Consortium (CPTAC) cancer cohorts (28). In our study, we applied the “LinkFinder” module to explore the correlated significant genes of AlkB family genes, and the “LinkInterpreter” module to perform the GO pathway enrichment analysis based on the association results.

### Immunohistochemistry

5 cases of normal brain and 20 cases of GBM formalin-fixed, paraffin-embedded (FFPE) tumor tissue blocks were submitted for immunohistochemistry (IHC). IHC was performed following standard protocols. The slides were dewaxed, boiled in 0.01 M citrate buffer (pH 6.0) for 30 minutes, and then incubated with 3% hydrogen peroxide for 15 minutes. After washing with PBS, the slides were incubated with 5% normal bovine serum albumin (BSA) for 1 hour, followed by incubation with rabbit polyclonal antibody recognizing ALKBH8 (A7142, 1: 100, ABclonal Technology, Wuhan, China) overnight. After incubating with secondary antibody, the slides were then incubated with 3,3′ diaminobenzidine (DAB) (PV-6000D, ZSGB-BIO, Beijing, China) for staining. The slides were then counterstained with hematoxylin, dehydrated, cleared, and mounted. The study was approved by the Ethics Committee of Xiangya Hospital (Ethics approval No. 202201015).

### Statistical Analyses

ALKBH8 expression profile difference between normal brain and GBM tissues was analyzed using Wilcoxon rank testing. *P*-value < 0.05 was considered to be statistically significant.

## Results

### Aberrant Expression of AlkB Family Members in GBM Patients

**Figure 1:**
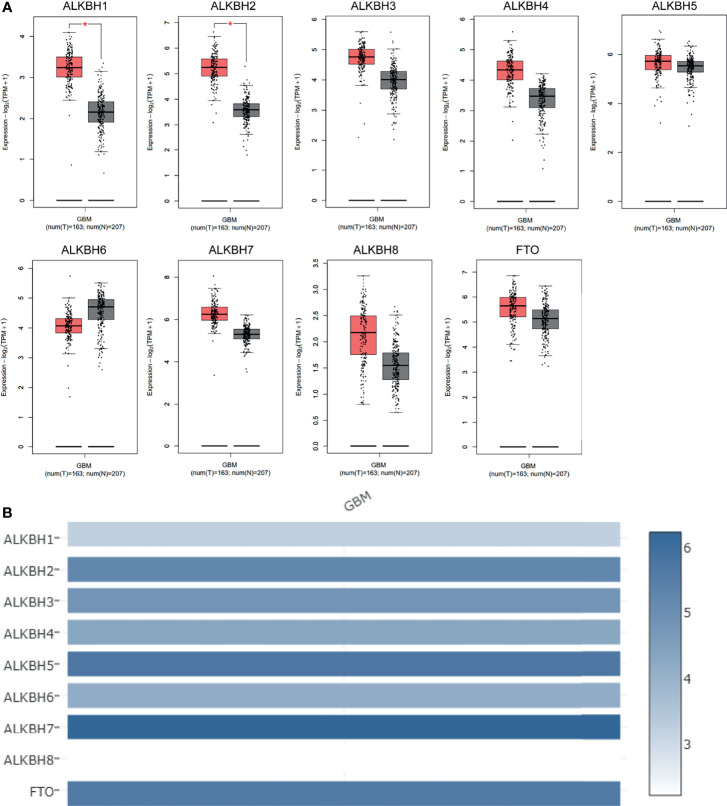
mRNA expression levels of the AlkB family members in GBM. **(A)** mRNA expression levels of the AlkB family members in GBM and normal brain tissues based on the GEPIA2 database. T and N indicated the tumor and normal tissues, respectively. **(B)** The relative mRNA expression levels of the AlkB family in GBM based on the GEPIA2 database. *p < 0.05.

**Figure 2:**
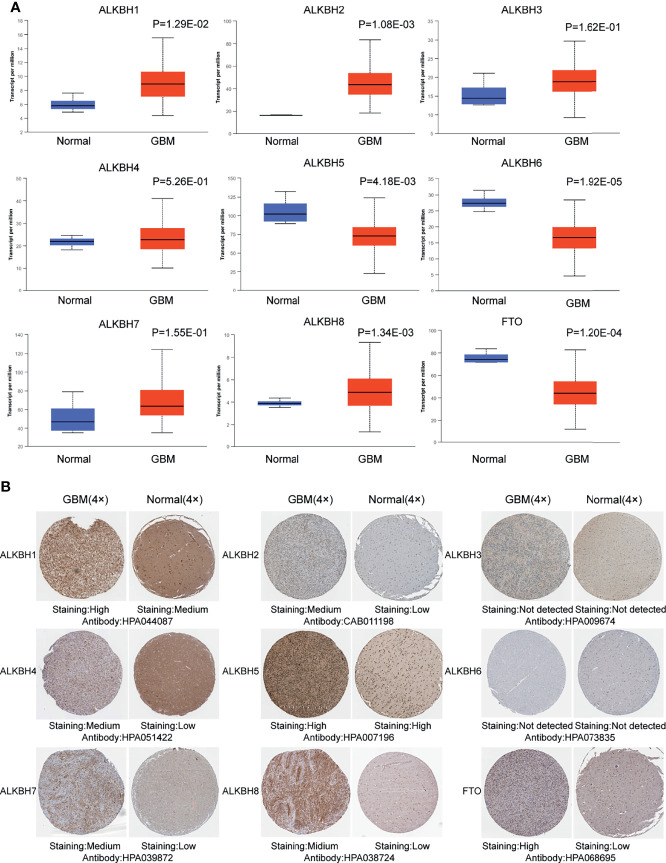
mRNA and protein expression levels of the AlkB family members in GBM. **(A)** mRNA expression levels of the AlkB family members in GBM and normal brain tissues by searching the UALCAN website. **(B)** Representative immunohistochemical staining of the AlkB family members in GBM and normal brain tissues based on the Human Protein Atlas database.

We firstly searched the GEPIA2 database to explore the mRNA expression levels of the AlkB family (ALKBH1-8 and FTO) in GBM and normal brain tissue. The results revealed that the expression levels of ALKBH1/2/3/4/5/7/8/FTO were elevated, while the expression level of ALKBH6 was reduced compared with normal brain tissues (**Figure 1A**). We also utilized GEPIA2 to analyze the relative expression level of the AlkB family in GBM, and the result illustrated that ALKBH5/7/FTO had relatively higher expression levels among the family members (**Figure 1B**). Besides the GEPIA2 database, we applied UALCAN to explore the mRNA expression levels of the AlkB family in GBM and normal brain tissue. The results showed that the expression levels of ALKBH1/2/8 were significantly up-regulated and the expression levels of ALKBH5/6/FTO were significantly down-regulated in GBM compared with normal tissues (**Figure 2A**). After description of the mRNA expression levels of AlkB family, we applied the Human Protein Atlas to explore the protein expression levels of AlkB family in GBM. The results revealed that ALKBH2/4/7/8/FTO were highly or mediumly expressed, while ALKBH3/6 were lowly or not detected in GBM tissues (**Figure 2B**). Taken together, these results indicated the potential biological roles of up-regulated ALKBH2/8 in GBM pathogenesis.

### Prognostic Value of AlkB Family mRNA Expression in GBM Patients

**Figure 3:**
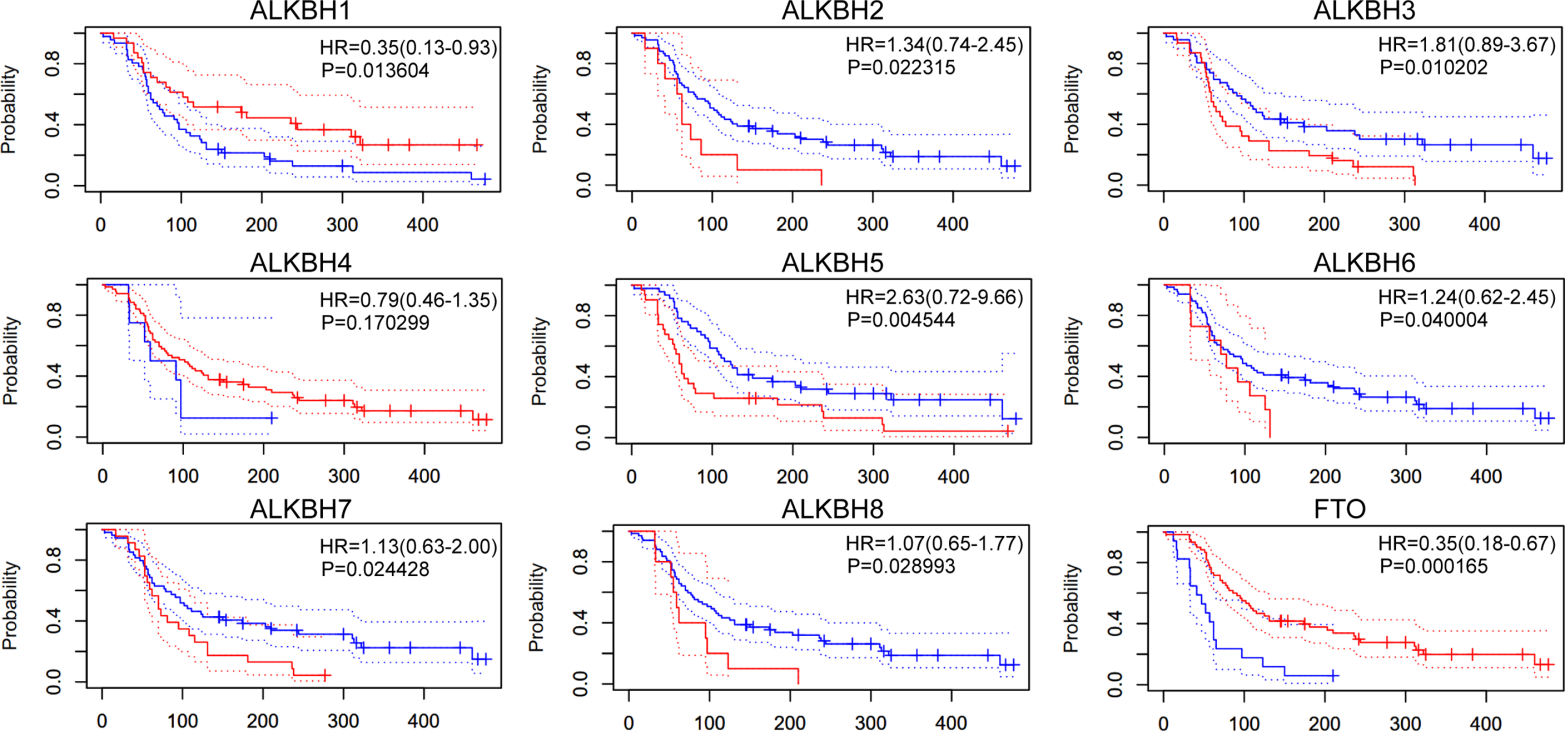
Overall survival of GBM patients. The overall survival of GBM patients based on the AlkB family genes expression in GEO GSE4271 dataset.

We next managed to investigate whether the mRNA expression levels of AlkB family genes were associated with GBM patients’ survival. After searching the PrognoScan database, we found intriguingly that patients with higher transcription levels of ALKBH2 and ALKBH8 possessed significant shorter overall survival (OS) time based on the GEO GSE4271 dataset (34) (**Figure 3**). Conversely, patients with higher transcription level of FTO displayed significant longer OS time.

### Genetic Alterations and Functional Analysis of the AlkB Family in GBM Patients

**Figure 4:**
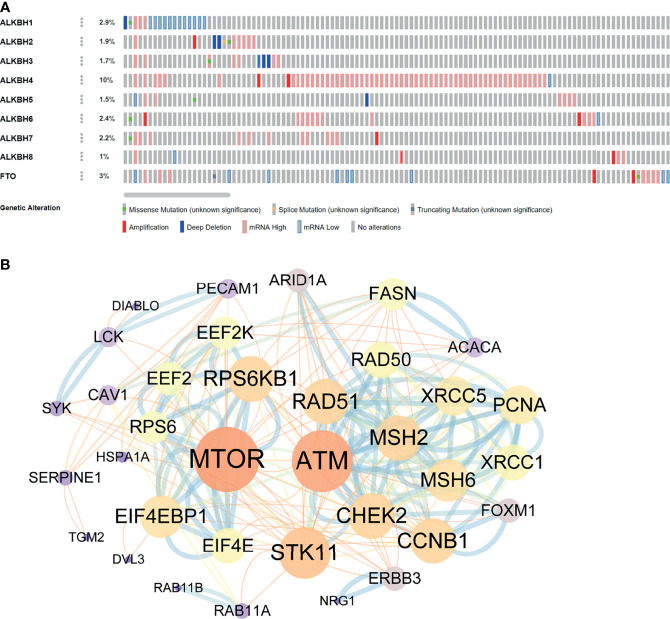
Genetic alterations of the AlkB family genes and related PPIs. **(A)** The genetic alterations of the AlkB family genes in GBM by searching the cBioPortal database. **(B)** The PPIs among the altered proteins most related to AlkB family genetic alterations generated by applying the STRING website and Cytoscape software.

**Figure 5:**
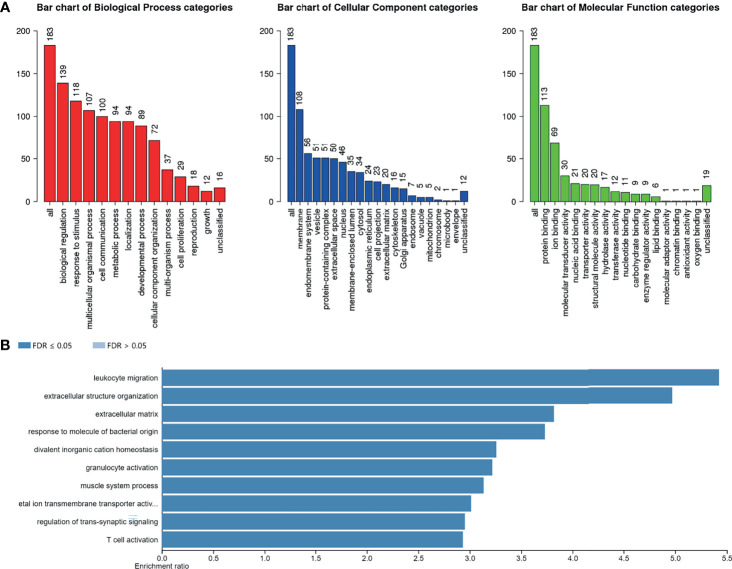
GO annotation and pathway enrichment analysis of the altered genes. **(A)** GO annotation of altered genes related to the AlkB family genetic alterations including biological process, cellular component and molecular function categories by applying the WebGestalt website. **(B)** GO pathway enrichment analysis of the altered genes by applying the WebGestalt website.

To furtherly explore the biological function of AlkB Family in GBM, we first evaluated the genetic alterations of the AlkB family genes by searching the cBioPortal database. The results presented that the genetic alteration rates of ALKBH1-8/FTO in GBM tumor samples were 2.9%, 1.9%, 1.7%, 10%, 1.5%, 2.4%, 2.2%, 1% and 3%, respectively (**Figure 4A**). We also found that “mRNA high” was the most common alteration mode for most of the AlkB family members. After confirming the genetic alteration profiling of AlkB family in GBM, we explored the altered proteins and genes in GBM samples with mutation of at least one gene of AlkB family by utilizing the cBioPortal database, and identified 38 proteins (**Supplementary Table 1**) and 183 genes (**Supplementary Table 2**) most related to AlkB family genetic alterations. Then we conducted protein interaction analysis by applying the STRING website and Cytoscape software to depict the PPIs among these 38 altered proteins based on the corresponding nodes and combined scores (**Supplementary Table 3**). We found that a variety of proteins displayed the critical functions determining cell fate. Specifically, a series of DNA damage repair related proteins, including ATM, CHEK2 and MSH2, were closely associated with the functions of AlkB family in GBM. Moreover, we also identified other proteins playing pivotal roles in controlling metabolism and immunity (**Figure 4B**). After analyzing the altered proteins, we performed GO annotation and pathway enrichment analysis by applying the WebGestalt website based on the 183 altered genes. The GO annotation results suggested that the altered genes were mainly associated with biological regulation, response to stimulus and metabolic process. Regarding cellular components, the altered genes were mostly enriched in membrane and endomembrane system. In terms of molecular function categories, the altered genes were mostly related to protein binding, ion binding and nucleic acid binding (**Figure 5A**). The corresponding enriched GO pathways included leukocyte migration, extracellular structure organization and T cell activation (**Figure 5B**).

### Immune Cell Infiltration Based on the AlkB Family Gene Expression in GBM Patients

**Figure 6:**
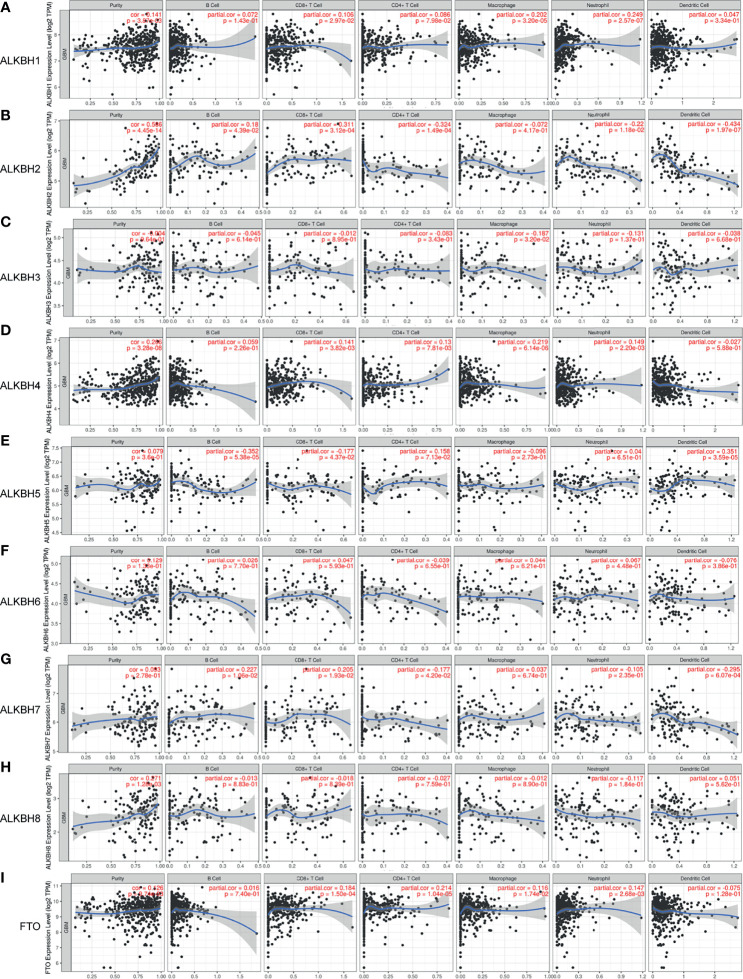
Correlation between the AlkB family gene expression and abundance of immune infiltrates in GBM. **(A-I)** The effect of ALKBH1 **(A)**, ALKBH2 **(B)**, ALKBH3 **(C)**, ALKBH4 **(D)**, ALKBH5 **(E)**, ALKBH6 **(F)**, ALKBH7 **(G)**, ALKBH8 **(H)**, and FTO **(I)** on the tumor immune cell infiltration analyzed by the TIMER database.

On account of the GO pathway enrichment results, we became interested in the correlation between AlkB family gene expression and abundance of immune infiltrates in GBM tissues. By searching the TIMER web server, we found that ALKBH1 expression level was positively correlated with CD8+ T cells, macrophages and neutrophils infiltration (**Figure 6A**). Regarding ALKBH2, its expression level was positively correlated with B cells and CD8+ T cells, and negatively correlated with CD4+ T cells, neutrophils and dendritic cells infiltration (**Figure 6B**). For ALKBH3, the only significant immune cells were macrophages, which was negatively correlated with the gene expression level (**Figure 6C**). Regarding ALKBH4, its expression level was positively correlated with CD8+ T cells, CD4+ T cells, macrophages and neutrophils (**Figure 6D**). For ALKBH5, its expression level was positively correlated with dendritic cells, and negatively correlated with B cells and CD8+ T cells infiltration (**Figure 6E**). Regarding ALKBH6, its expression level was not significantly tied to immune cell infiltration (**Figure 6F**). For ALKBH7, its expression level was positively correlated with B cells and CD8+ T cells, and negatively correlated with CD4+ T cells and dendritic cells infiltration (**Figure 6G**). Regarding ALKBH8, its expression level was not significantly related to immune cell infiltration (**Figure 6H**). For FTO, its expression level was positively correlated with CD8+ T cells, CD4+ T cells, macrophages and neutrophils, which suggested active anti-tumor microenvironment and might explain patients with higher transcription level of FTO possessed significant longer OS time (**Figure 6I**). After analyzing the correlation between AlkB family gene expression and immune infiltrates in GBM tissues, we furtherly assessed the association of clinical outcome with immune cell infiltration and the AlkB family gene expression by applying the multivariable Cox proportional hazard model. The results revealed that CD4+ T cells, dendritic cells and ALKBH4/6/8 were significantly associated with patient survival (**Table 2**).

**Table 2:** The association of clinical outcome of GBM patients with immune cell infiltration and the AlkB family gene expression by applying the multivariable Cox proportional hazard model from the TIMER database.

	coef	HR	95%CI_I	95%CI_u	*P* value	sig
B cell	0.388	1.474	0.235	9.258	0.679	
CD8_Tcel	0.512	1.668	0.485	5.734	0.417	
CD4_Tcell	3.261	26.088	2.421	281.060	0.007	**
Macrophage	-1.035	0.355	0.026	4.917	0.440	
Neutrophil	-1.396	0.248	0.011	5.712	0.383	
Dendritic	2.075	7.967	2.265	28.029	0.001	**
ALKBH1	-0.008	0.992	0.485	2.032	0.983	
ALKBH2	-0.175	0.839	0.390	1.807	0.655	
ALKBH3	0.189	1.208	0.606	2.408	0.591	
ALKBH4	0.973	2.646	1.279	5.475	0.009	**
ALKBH5	0.147	1.158	0.585	2.293	0.674	
ALKBH6	0.821	2.272	1.211	4.260	0.011	*
ALKBH7	-0.115	0.891	0.444	1.789	0.746	
ALKBH8	-0.869	0.419	0.190	0.927	0.032	*
FTO	0.045	1.047	0.492	2.226	0.906	

*p < 0.05, **p < 0.01.

### Correlated Significant Genes and Corresponding Enriched GO Pathways of ALKBH2/8

**Figure 7:**
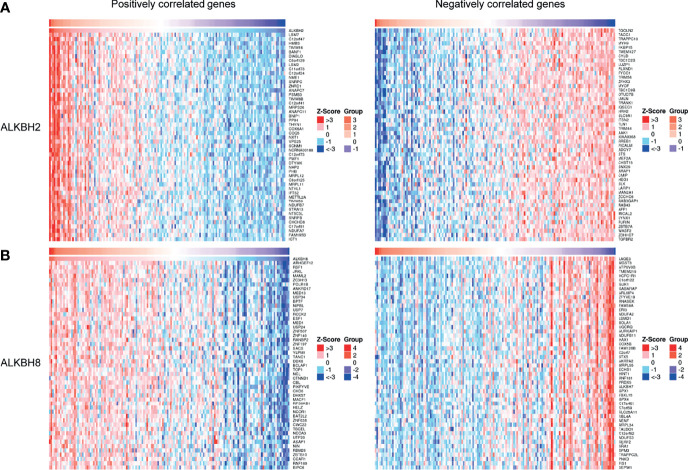
Correlated significant genes of ALKBH2/8 in GBM. **(A, B)** The positively and negatively correlated significant genes of ALKBH2 **(A)** and ALKBH8 **(B)** by taking advantage of the LinkedOmics website.

**Figure 8:**
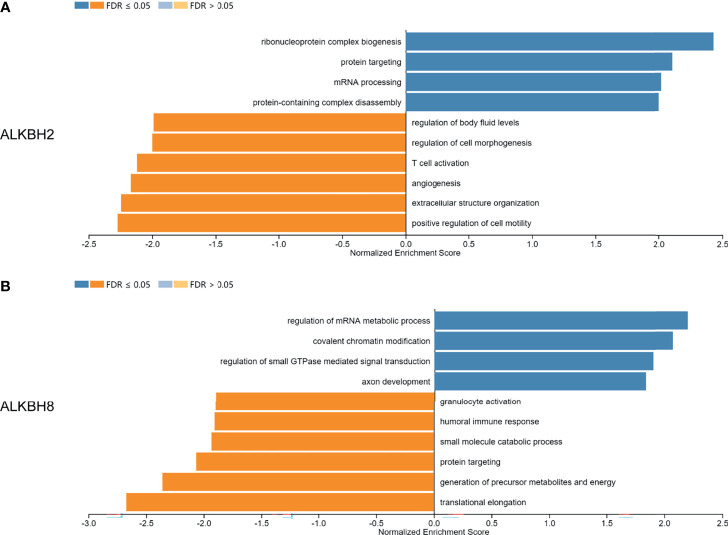
GO pathway enrichment analysis based on the correlated genes of ALKBH2/8 in GBM. **(A, B)** The GO pathway enrichment results based on the correlated genes of ALKBH2 **(A)** and ALKBH8 **(B)** by taking advantage of the LinkedOmics website.

After identifying ALKBH2/8/FTO as significant prognostic biomarkers based on the mRNA expression levels, we proceeded to explore the correlated significant genes of ALKBH2/8, and to perform the GO pathway enrichment analysis based on the association results by taking advantage of the LinkedOmics website. The positively and negatively correlated significant genes were exhibited in **Figures 7A, B**. We noticed that the correlated genes consisted of DNA damage repair related genes such as NTHL1, PCNA and ERCC8, and cell metabolism related genes such as MRPS26, EIF5A and EEF1G, which was consistent with the previous results. Concerning the GO pathway enrichment results, we found that mRNA processing was positively correlated with the expression level of both ALKBH2 and ALKBH8, and immune response was negatively correlated with ALKBH2/8 expression level, which might partly explain the worse survival of GBM patients with higher transcription levels of ALKBH2/8 (**Figures 8A, B****)**. The high expression of ALKBH8 in GBM was verified by IHC.

**Figure 9:**
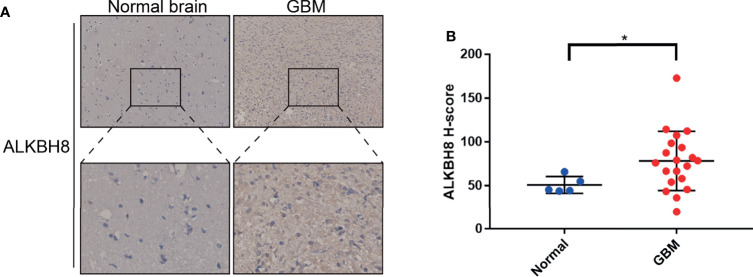
Representative IHC images and H-score of ALKBH8 protein expression in GBM and normal brain tissues. **(A)** Immunohistochemical staining for ALKBH8 in human GBM and normal brain tissues. The specific ALKBH8 signal is shown in brown (DAB staining). **(B)** The difference of ALKBH8 protein expression between GBM and normal brain was assessed by Wilcoxon rank testing. *p < 0.05.

After analyzing the online databases, we performed the ALKBH8 staining in 5 normal brain tissues and 20 GBM tissues. The IHC results illustrated that ALKBH8 was highly expressed in GBM tissue compared with normal brain, which is consistent with the results obtained from the databases (**Figures 9A, B****)**.

## Discussion

The AlkB family members possess a variety of important biological functions and play significant roles in the development of tumor including GBM. By generating the genetic alteration analysis and associated altered proteins interaction interpretation, we identified a series of DNA damage repair related proteins such as ATM and CHEK2, suggesting the critical role of this family played in GBM. As a DNA repair enzyme, ALKBH2 functions through removing alkyl substituents from alkylation-damaged nucleic acid bases. It was reported that ALKBH2 was abundantly expressed in GBM cell lines and human GBM tissues, and enhanced resistance to the alkylating agents TMZ (20). Another study suggested ALKBH2 expression was implicated in resistance to photodynamic therapy in U87 GBM cells (19). Though the potential roles of this family members in cancer are rapidly uncovered, the comprehensive function of this particular family in GBM has not been elucidated yet.

Besides the DNA damage repair related proteins, we also found mTOR and its downstream effector molecules such as EIF4E and RPS6 were closely related to the AlkB family genetic alterations. In eukaryotes, mTOR signaling pathway integrates both intracellular and extracellular signals and serves as a central regulator of cellular metabolism and survival (35, 36). Moreover, major DNA damage sensors such as ATM, ATR and DNA-PK communicate with the mTORC1 pathway to ensure an efficient response to the metabolic and genotoxic stresses (37). Beuvink et al. reported that mTOR inhibitor everolimus sensitizes tumor cells to DNA-damaged induced apoptosis (38). Another study suggested that m6A RNA modification catalyzed by enzymes including ALKBH5 and FTO had a fundamental role in the regulation of PI3K/Akt/mTOR signaling pathway in cancer (39). HT Zhu et al. revealed that ALKBH5 was upregulated and activated EGFR-PIK3CA-AKT-mTOR signaling pathway in epithelial ovarian cancer tissue (40). Yet, the crosstalk between AlkB family members and the metabolic pathways in GBM need to be furtherly elucidated.

Aberrant epigenetic landscapes largely affected tumor initiation and progression, and The AlkB family was continuingly studied within this aspect. DNA methylation is a critical epigenetic mark which plays a fundamental role in cancer. Xie et al. demonstrated that ALKBH1 was a critical demethylase for DNA N6-mA and regulated specific genetic pathways in GBM. Depletion of ALKBH1 could cause transcriptional silencing of oncogenic pathways through decreasing chromatin accessibility and inhibit tumor cell proliferation (41). Besides DNA modifications, RNA processing also plays major roles in cells and tumors. N6-Methyladenosine (m6A) is the most prevalent internal chemical modification of mRNAs in eukaryotes, which can affect the stability and translation efficiency of mRNA (42–44). Our results suggested that mRNA processing was positively correlated with the expression level of both ALKBH2 and ALKBH8 based on the GO pathway enrichment analysis. Zhang et al. revealed that ALKBH5, a m6A demethylase, was highly expressed in glioblastoma stem-like cells (GSCs) and demethylated FOXM1 nascent transcripts leading to enhanced FOXM1 expression. Their work suggested ALKBH5-FOXM1 pathway was critical for GSC proliferation and tumorigenesis, and uncovered the critical function of ALKBH5 in GBM (45). FTO is another frequently studied RNA m6A demethylase. Rui Su et al. reported that R-2HG displayed anti-tumor activity in leukemia and glioma through targeting FTO, leading to MYC mRNA decay and downregulation of MYC/CEBPA-associated oncogenic pathways (46).

Accumulating evidence suggests that the tumor immune microenvironment plays a critical role in anti-cancer immunity, which may affect patient prognosis and the effect of immunotherapy (47, 48). Our results suggested significant correlation between AlkB family gene expression and abundance of immune infiltrates in GBM tissues based on the TIMER website, and the corresponding enriched GO pathways included leukocyte migration and T cell activation associated with the family genes genetic alterations. In addition to the significant roles of RNA m6A modifications in tumor-intrinsic oncogenic pathways, increasing evidence supports that RNA m6A metabolism also functions in tumor immune regulation (49, 50). Li and his colleague reported that ALKBH5 modulated Mct4/Slc16a3 expression and lactate content of the tumor microenvironment and promoted the composition of tumor-infiltrating Treg and myeloid-derived suppressor cells in melanoma and colorectal cancer, thus leading to resistance to immunotherapy. And deletion of ALKBH5 sensitized tumors to cancer immunotherapy (51). Another study revealed that FTO expression level was increased in human melanoma and contributed to promoting melanoma tumorigenesis and anti-PD-1 resistance. Knockdown of FTO could sensitize melanoma cells to interferon gamma and anti-PD-1 treatment in mice (52). However, the relevance between AlkB family members and tumor immunity in GBM need to be furtherly explored.

We are aware of the limitations of this study. Firstly, the study mainly contains results from the public database, but is short of experimental verification. Though the expression of ALKBH8 in GBM was verified by IHC, more *in vitro* and *in vivo* experiments are needed to explore the biological function of this family in GBM. Secondly, the data sets used in different databases are inconsistent, and some contradictory data need to be further confirmed. Thirdly, we need further research to verify the therapeutic significance of AlkB family members in GBM patients.

In conclusion, by taking advantage of the abundant bioinformatics databases, we conducted this comprehensive analysis of the AlkB family in GBM for the first time, and managed to identify the value of evaluating patients’ prognosis based on the mRNA expression levels of ALKBH2/8/FTO. In addition, we revealed that the biological function of the AlkB family was closely related to DNA damage repair, cell metabolism, cell proliferation and tumor immune infiltration in GBM, which needed to be furtherly explored in the future. We believe that the existing results could help researchers discover novel mechanisms associated with GBM initiation and progression, and help clinicians precisely predict patient survival and select alternative therapeutic drugs.

## Data Availability Statement

The original contributions presented in the study are included in the article/**Supplementary Material**. Further inquiries can be directed to the corresponding authors.

## Ethics Statement

The studies involving human participants were reviewed and approved by Ethics Committee of Xiangya Hospital. Written informed consent for participation was not required for this study in accordance with the national legislation and the institutional requirements.

## Author Contributions

Acquisition of Data: SF and ZX. Analysis and Interpretation of Data: ZX and SF. Conception and Design: ZX and JP. Data Curation: SF. Development of Methodology: ZX. Performing the experiment: MZ. Writing the Manuscript: SF, JP, and MZ. All authors contributed to the article and approved the submitted version.

## Funding

This study was supported by the National Natural Science Foundation of China (No. 82102848) and the Hunan Provincial Natural Science Foundation of China (No. 2020JJ8111).

## Conflict of Interest

The authors declare that the research was conducted in the absence of any commercial or financial relationships that could be construed as a potential conflict of interest.

## Publisher’s Note

All claims expressed in this article are solely those of the authors and do not necessarily represent those of their affiliated organizations, or those of the publisher, the editors and the reviewers. Any product that may be evaluated in this article, or claim that may be made by its manufacturer, is not guaranteed or endorsed by the publisher.
